# An innovative staged prosthetic lengthening reconstruction strategy for osteosarcoma-related leg discrepancy

**DOI:** 10.1038/s41598-023-50422-8

**Published:** 2024-01-06

**Authors:** Hairong Xu, Yuan Li, Feng Yu, Weifeng Liu, Lin Hao, Qing Zhang, Xiaohui Niu

**Affiliations:** JST Sarcoma & Bone Tumor Center, Beijing Jishuitan Hospital, Capital Medical University, Beijing, China

**Keywords:** Surgical oncology, Bone cancer

## Abstract

Correction of leg length discrepancy (LLD) in skeletally mature patients with osteosarcoma was rarely reported and quite challenging. This study aimed to propose a treatment strategy of staged lengthening and reconstruction with a standard static prosthesis to address LLD and restore limb function. It also evaluated the effectiveness of the strategy in terms of leg lengthening, functional outcomes, and complications. The strategy for lengthening included three stages. In stage 1, the previous prosthesis was removed and an external fixator with a temporary rod-cement spacer was placed. In this stage, the external fixator was used to lengthen the limb to the appropriate length. In stage 2, the external fixator was removed and the old rod-cement spacer was replaced with a new one. In stage 3, the rod-cement spacer was removed and the standard static prosthesis was planted. Nine skeletally mature distal femoral osteosarcoma patients with unacceptable LLD were treated in our institution from 2019 to 2021. We performed a chart review on nine patients for the clinical and radiographic assessment of functional outcomes, LLD, and complications. The mean (range) leg lengthening was 7.3 cm (3.6–15.6). The mean (range) LLD of the lower limbs decreased from 7.6 cm (4.1–14.2) before the lengthening to 0.3 cm (− 0.3 to 2.1) at the final follow-up with statistical significance (*P* = 0.000). The mean (range) Musculoskeletal Tumor Society score improved from 30.3% (16.7%–53.3%) before the lengthening to 96.3% (86.7%–100%) at the final follow-up with statistical significance (*P* = 0.000). Three patients (33.3%) had a minor complication; none needed additional surgical intervention. In the short term, the current staged lengthening and reconstruction with standard static prosthesis provided satisfactory functional outcomes and LLD correction with few complications. The long-term effects of this method need further exploration.

## Introduction

Osteosarcoma is the most common primary malignant bone tumor in children and adolescents, occurring in 2–3 per million individuals a year^1^. About 44% of the tumors occur in the distal femur^2^. Tumor resection with adequate margin and prosthesis reconstruction is the mainstay of surgical treatment. Nevertheless, it may be difficult to spare the physis due to oncologic reasons in skeletally immature cases, and hence leg length discrepancy (LLD) develops for patients with long-term survival^3–5^. In both skeletally mature and immature patients, aseptic loosening and subsequent subsidence of prosthesis could result in LLD as well^6^. LLD is often accompanied by poor walking gait and extremity function, which negatively impacts the quality of life in patients^7^. LLD can cause low back pain and hip osteoarthritis in the long term.

Several surgical options have been reported to prevent and treat LLD in skeletally mature patients with distal femoral osteosarcoma. Expandable prosthesis, designed for skeletally immature patients in 1976^4^, has greatly evolved over decades^6,8,9^. Yet the complication rate of expandable prosthesis remains higher than that of the standard static prosthesis^10,11^, and only a few cases have been reported for skeletal mature patients^12,13^. Besides, expandable prostheses are not available in China currently. Some authors proposed temporary hemiarthroplasty or arthrodesis^14,15^, followed by staged distraction osteogenesis lengthening and standard prosthesis replacement. Distraction osteogenesis was originally proposed as an option for large diaphyseal defects. Its accompanying complications limit its wide application in oncological patients. We proposed a strategy of staged lengthening and reconstruction with a standard static prosthesis to solve the LLD and lower the potential procedure-associated complications. The lengthening focused on the soft tissue, not on the bone.

In this study, we addressed the following questions: (1) What were the results with respect to the change in LLD? (2) What were the Musculoskeletal Tumor Society (MSTS) scores after using this lengthening strategy was used? (3) What complications occurred during and after this lengthening strategy was used?

## Patients and methods

### Clinical series

The research protocol had been approved by the Institutional Ethics Committee of Beijing Ji Shui Tan Hospital, Beijing, China. Informed consent was obtained from all participants and/or their legal guardian(s), and methods were carried out in accordance with relevant guidelines and regulations.

Patients were considered for staged lengthening and reconstruction with a standard static prosthesis if all of the following criteria were met: (1) skeletally mature patient; (2) LLD ≥ 4 cm; (3) expected long-term survival; and (4) standard prosthesis reconstruction possible. The exclusion criteria were as follows: (1) skeletally immature patient; (2) LLD < 4 cm; (3) metastatic osteosarcoma; and (4) poor response to neoadjuvant chemotherapy.

Based on the aforementioned indications, nine patients were treated with staged lengthening and reconstruction for unacceptable LLD between 2019 and 2021. The study included two male and seven female patients. The mean age at the lengthening surgery was 21 years (range, 14–35). All patients were previously diagnosed with osteosarcoma in the distal femur. Eight of nine patients were primarily treated in our institution. The remaining one patient, who was previously treated with inactivated autograft replantation, was referred to us for the treatment of LLD.

**Figure 1:**
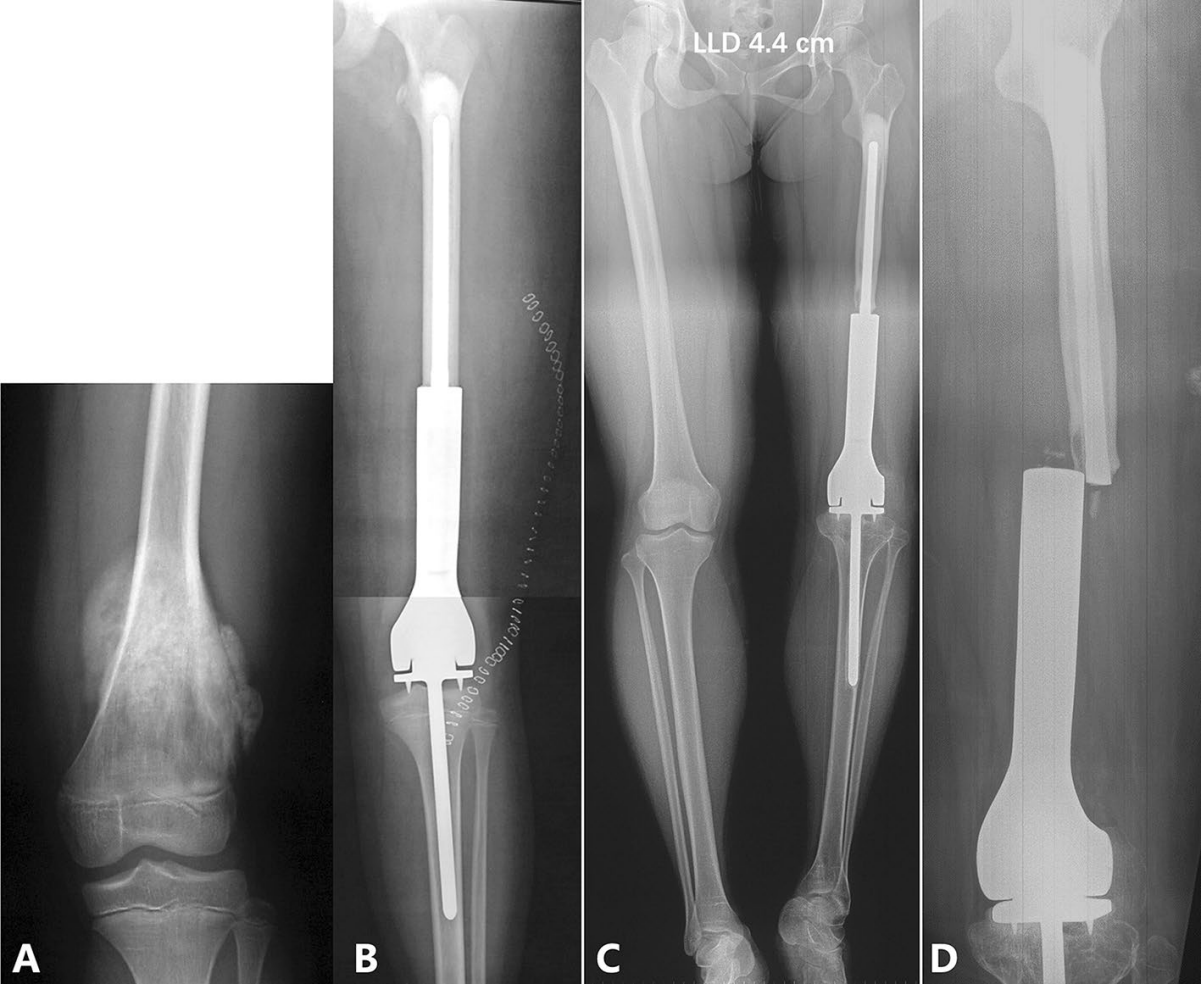
A 10-year-old girl with left femoral osteosarcoma was treated with chemotherapy and prosthesis replacement (patient no. 2 in Table 1). (**A**) A radiograph shows that the tumor was located in the left distal femur. (**B**) After tumor resection, the prosthesis replacement was done while preserving the tibia physis. (**C**) The LLD of the lower limbs was 4.4 cm, 13 years after the primary tumor surgery. (**D**) Fourteen years after the primary tumor surgery, the patient fell and the radiograph showed a breakage of the prosthesis.

The most common reason for LLD was prosthesis replacement for skeletally immature patients. When they grew up, the LLD developed. Six patients fell into this category. This subset included two patients with prosthesis breakage and one patient with aseptic loosening (Fig. 1). Other reasons for LLD included aseptic loosening and prosthesis breakage (adult), inactivated autograft collapse, and pathological fracture of primary femoral osteosarcoma (Table 1).

**Table 1:** Patients demographics.

Patient No	Gender	Age at resection (years)	Age at staged lengthening (years)	Reasons for LLD	Original tibial prosthesis component removed	Final prosthesis
1	M	16	33	Prosthesis aseptic loosening*	Yes	DFP
2	F	10	24	PR-SIP*	Yes	DFP
3	F	9	20	PR-SIP	No	DFP
4	F	9	16	PR-SIP	Yes	DFP
5	F	10	14	PR-SIP*	No	DFP
6	F	12	21	PR-SIP	Yes	TFP
7	F	18	18	Pathological fracture of primary tumor	Yes	DFP
8	F	14	24	PR-SIP#	Yes	TFP
9	M	33	35	Inactivated autograft collapse	NA	DFP

PR-SIP: Prosthesis replacement for skeletal immature patients, DFP: Distal femoral prosthesis, TFP: Total femoral prosthesis, *Complicated with prosthesis breakage, #Complicated with aseptic loosening.

### Treatment strategy

**Figure 2:**
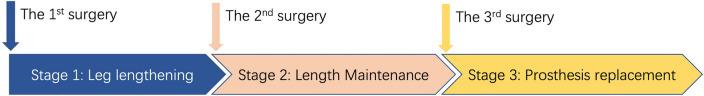
Flow chart showing the treatment strategy of staged lengthening and reconstruction of the standard static prosthesis.

Our strategy for lengthening included three stages (Fig. 2). In stage 1, the previous prosthesis was removed and an external fixator with a temporary rod-cement spacer was placed (the first surgery). The objective of this stage was to correct the LLD by lengthening the affected thigh. In stage 2, the external fixator was removed, and the old rod-cement spacer was replaced with a new one (the second surgery). The objective was to maintain the corrected leg length while reducing the potential prosthesis infection. In stage 3, the rod-cement spacer was removed, and the standard static prosthesis was planted (the third surgery). The objective of this stage was to recover regular limb function.

**Figure 3:**
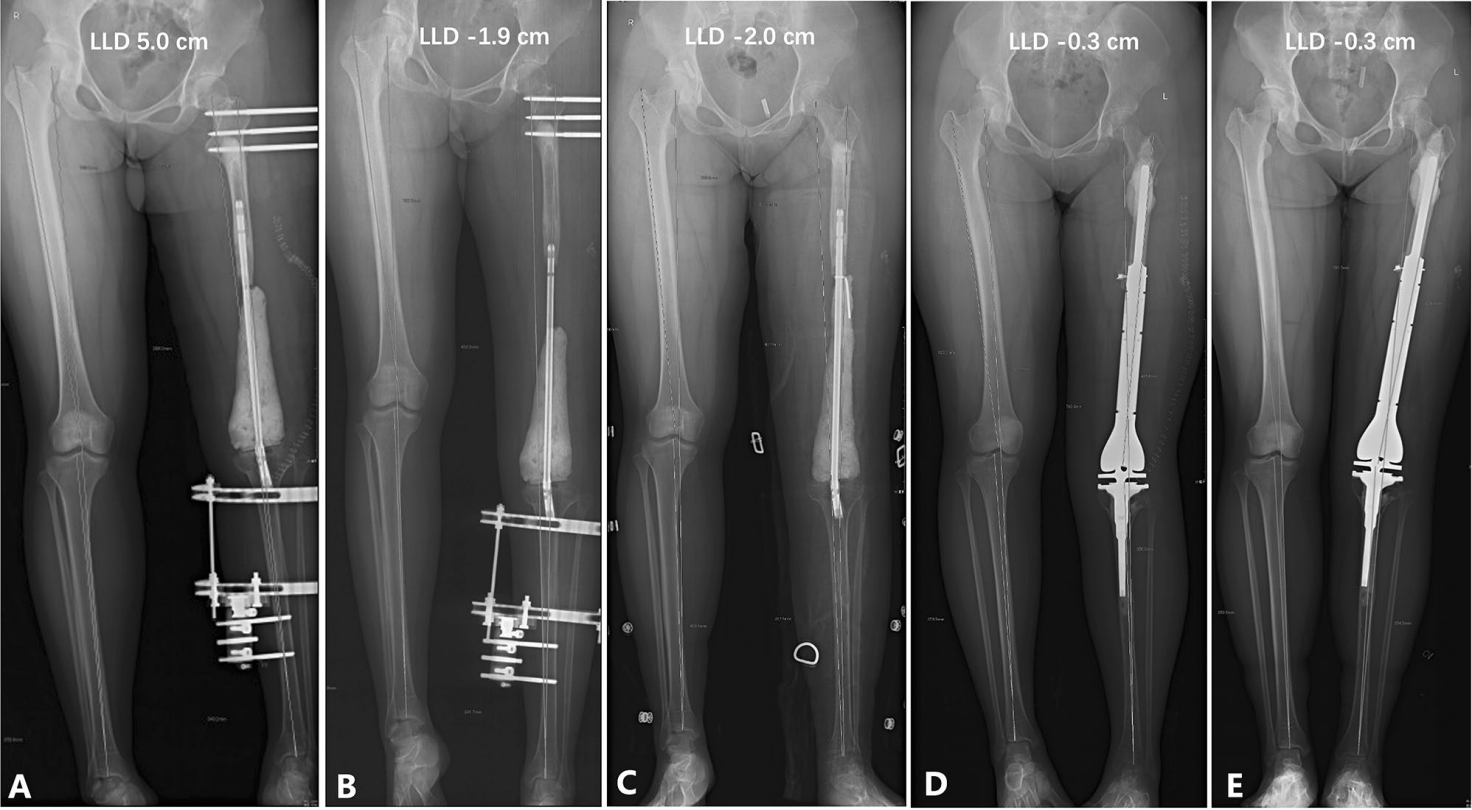
This was the same patient as in Fig. 1 (patient 2 in Table 2). (**A**) A radiograph showed that the LLD was 5.0 cm immediately after the first surgery in stage 1. (**B**) A radiograph showed that the LLD was overcorrected by 1.9 cm at the end of stage 1. (**C**) A radiograph showed that the LLD was − 2.0 cm during stage 2. (**D**) A radiograph showed that the LLD was − 0.3 cm immediately after the third surgery in stage 3. (**E**) Twenty-five months after the final surgery, the radiograph showed that the LLD did not change over time.

In stage 1, the external fixator was used to lengthen the soft tissue, not the bone. The discrepancy was planned to be overcorrected by approximately 2 cm (Fig. 3A,B). The lengthening was started on the third postoperative day at a rate of 0.5 mm twice a day. It could be delayed if the patient developed pain or unacceptable neurological symptoms. Early ankle motion and quadriceps exercise were encouraged 2 days postoperatively as tolerated. From stage 1 to stage 2, to prevent potential osteoporosis, weight-bearing was maintained at 5–10 kg, not exceeding 15 kg at most. During this period, X-rays were taken every 8 weeks to monitor the position of the fixator and the length of the lower limb. In stage 2, the temporary rod and cement were used to maintain the leg length and promote soft tissue recovery. Early rehabilitation of ankle and quadriceps was encouraged as in stage 1 (Fig. 3C). A knee brace was applied to stabilize the knee joint 2 weeks postoperatively, and patients were allowed to walk with two crutches. From stage 2 to the stage 3, weight-bearing was maintained at 10–20 kg, not exceeding 25 kg at most. During this period, X-rays were taken every 12 weeks to monitor the position of the implant. In this stage, it was necessary to regularly monitor the blood routine, erythrocyte sedimentation rate, and C-reactive protein, and pay attention to the maintenance of needle eyes to avoid infection.

In stage 3, the shortening of approximately 2 cm of the femoral length and the release of excessive soft tissue around the knee may be needed to ensure the range of motion (ROM) and the coverage of the mega prosthesis. The discrepancy should be corrected as both limbs with equal lengths (less than 2 cm) (Fig. 3D,E). Prosthetic component lengths were planned preoperatively, but some margin for error was required. Intraoperatively, the length of the prosthesis was also adjusted according to the soft tissue tension. The knee ROM should be at least 120 degrees during the surgery. The patients were encouraged to perform range-of-knee movement exercises 2 weeks postoperatively. Partial weight-bearing was allowed 4–6 weeks postoperatively and full weight-bearing 3 months postoperatively. X-rays were taken every 3–6 months.

### Other treatment specifics and oncological outcome

The mean (range) duration of lengthening in stage 1 was 105 days (66–184). The mean (range) lengthening of the lower limb was 0.7 mm (0.5–1.0) per day. The mean duration from the first surgery in stage 1 to the second surgery in stage 2 was 5.5 months (2.3–10.1). The mean duration from the second surgery in stage 2 to the third surgery in stage 3 was 6.1 months (3.1–11.7). The mean (range) duration from the first surgery to the third surgery was 12 months (7–14). The mean (range) follow-up was 131 months (21–246) (from the initial primary tumor surgery) and 20 months (3–70) (from the third surgery in stage 3).

**Figure 4:**
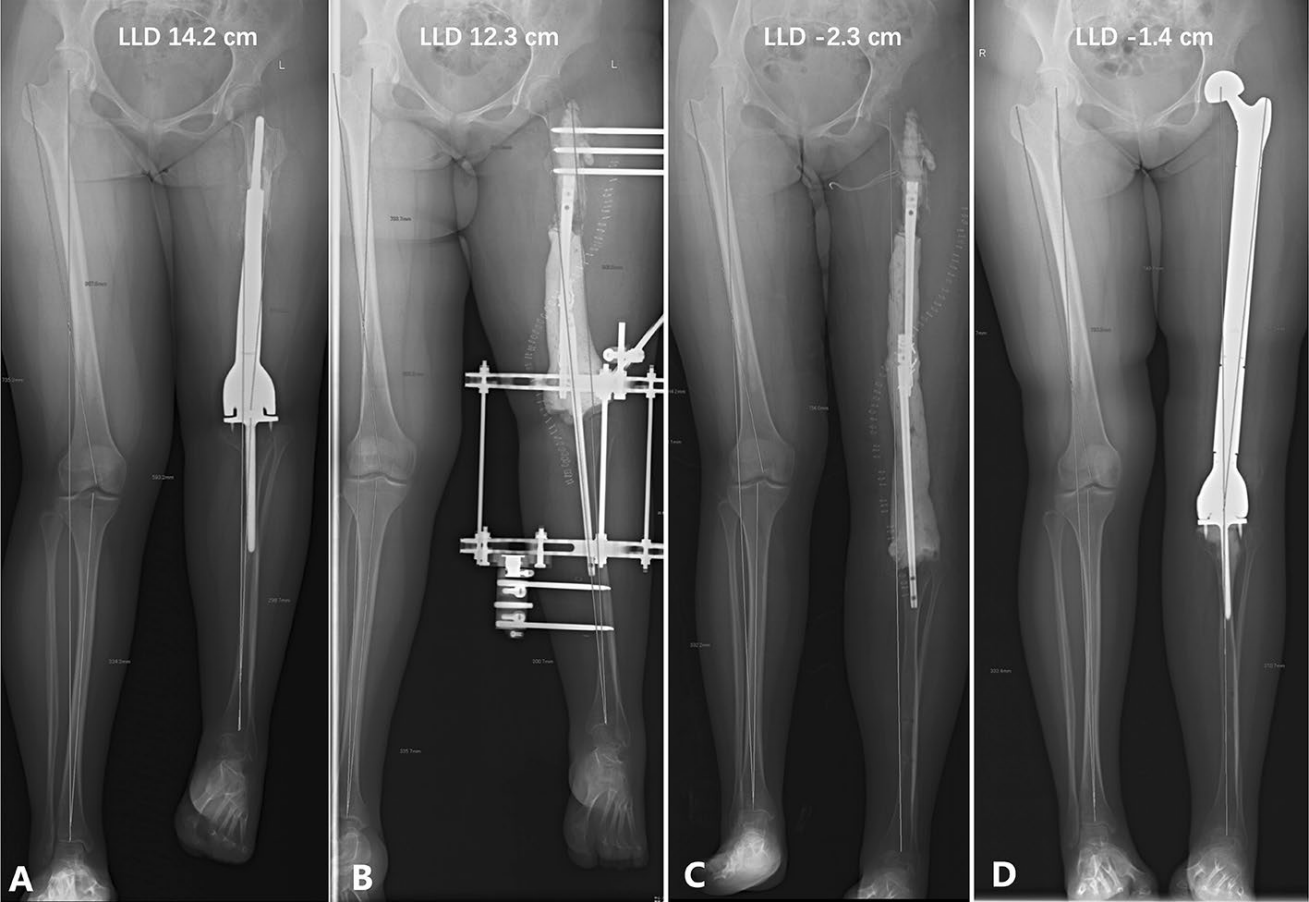
A 21-year-old woman with left femoral osteosarcoma was treated with chemotherapy and prosthesis replacement when she was 12 years old (patient no. 6 in Table 1). (**A**) The LLD of the lower limbs was 14.2 cm. (**B**) A radiograph showed that the LLD was 12.3 cm immediately after the first surgery in stage 1. (**C**) A radiograph showed that the LLD was overcorrected by 2.3 cm. (**D**) A radiograph showed that the total femoral replacement was down, and the LLD was − 1.4 cm immediately after the third surgery in stage 3.

During the first surgery, the tibial prosthesis was not removed in two patients. This was because the prosthesis and the bone were healed through bone ingrowth rather than bone cement. The prosthesis was difficult to remove and hence not removed. During the third surgery, seven patients underwent standard distal femoral mega-prosthesis replacement. The remaining two patients underwent total femur mega-prosthesis replacement due to the limited length and host bone quality of residual bone in the proximal femur (Fig. 4).

No patient developed local recurrence or distant metastasis during and after the staged lengthening surgeries.

### Functional and complication evaluation

The leg length was measured three times to evaluate the change of LLD; before soft tissue lengthening, after soft tissue lengthening, and at the final follow-up. The LLD was measured using full-length standing radiographs from the center of the femoral head to the center of the ankles of both lower limbs. The tibia length discrepancy (TLD) of both limbs was measured before the first surgery and at the last follow-up.

To answer the question on functional outcome, we performed a chart review on the nine patients to assess MSTS scores before the first surgery and at the final follow-up visit. We recorded the subsets of the MSTS score, including pain, function, emotional acceptance, support, walking, and gait. The knee ROM was recorded before the first surgery and at the final follow-up visit. ROM was used to evaluate the possible flexion contracture due to the treatment of the external fixator.

We recorded complications during the lengthening and after prosthesis reconstruction to answer the third question. These complications required removal or revision of a component; infection, aseptic loosening, or permanent functional impairment, such as a neurovascular injury or amputation, were classified as major complications^16^. Minor complications included osteoporosis, transient nerve palsy, or other events requiring no additional surgery.

### Ethical approval

The ethical approval for this study was obtained from *** Hospital.

### Statistical analysis

For quantitative variables, we reported the mean and range. The categorical variables were reported as counts. We performed a paired-sample *t* test to compare the values between two time points. We performed all statistical analyses using a commercially available software package (IBM SPSS Statistics for Mac, version 25, IBM Corp.). A *P* value of < 0.05 indicated a statistically significant difference in all tests.

## Results

### Leg length discrepancy

The mean (range) leg lengthening was 7.3 cm (3.6–15.6). The mean (range) LLD after lengthening (immediately after the second surgery) was − 1.4 cm (–3.1 to 2). The mean (range) LLD of the lower limbs decreased from 7.6 cm (4.1–14.2) before the lengthening to 0.3 cm (− 0.3 to 2.1) at the final follow-up with statistical significance (paired-sample *t* test: *t* = 6.263; *P* = 0.000). The mean (range) femur and TLD was –1.3 cm (− 3.6 to 1.1) and 1.3 cm (0.1–2.3) respectively, indicating that the knee center did not change significantly (Table 2).

**Table 2:** Length discrepancy change in leg, femur, tibia.

Patient No	Leg length discrepancy (cm)*			Femur length discrepancy (cm) *		Tibia length discrepancy (cm) *	
	Before lengthening	After lengthening	At final follow-up	Before lengthening	At final follow-up	Before lengthening	At final follow-up
1	6.5	− 0.9	1.1	5.4	− 0.3	1.5	1.5
2	5.0	− 1.9	− 0.3	3.9	− 2.4	2.0	1.5
3	7.9	− 3.1	2.1	5.8	− 0.1	1.9	1.9
4	8.6	− 0.6	1.0	6.9	− 0.9	1.8	1.8
5	4.3	− 2.9	0.7	4.8	− 2.8	1.4	1.4
6	14.2	− 2.3	− 1.4	11.7	− 3.6	2.3	2.3
7	4.1	− 1.5	− 1.2	4.1	− 1.4	0.7	0.7
8	9.5	2.0	1.2	9.7	1.1	0.1	0.1
9	8.3	− 1.6	− 0.3	7.4	− 1.3	1.1	1.1

*Discrepancy was obtained by subtracting the length of the affected limb from the length of the healthy limb.

### Functional outcomes

The mean (range) MSTS score of all nine patients improved from 30.3% (16.7%–53.3%) before the lengthening to 96.3% (86.7%–100%) at the final follow-up with statistical significance (paired-sample *t* test: *t* = − 15.8; *P* = 0.000). All nine patients were pain free and emotionally enthused, with no support needed at the last follow-up. Eight patients showed no restricted function. One patient showed intermediate function between no restriction and recreational restriction, and intermediate gait between normal and minor. Two patients with total femoral prosthesis replacement showed gluteal gait with intermediate walking (between unlimited and limited).

The mean (range) ROM before the lengthening and at the final follow-up was 98.3 degrees (20–135) and 77.8 degrees (55–90), respectively. The difference between the two time points was not statistically significant (paired-sample *t* test: *t* = − 1.2; *P* = 0.253). The ROM improved in two patients; one was with a primary pathological femoral fracture and the other was with inactivated autograft failure. The ROM decreased in the remaining seven patients. All nine patients showed the subset of the MSTS score “emotional acceptance” as enthused at the last follow-up.

### Complications

Two patients had osteoporosis before the third surgery, but both recovered 3 months after the third surgery. One patient forgot to wear his knee brace, and genu valgum developed during stage 2. Pin site infection occurred in one patient (complicated with osteoporosis as well) during the leg lengthening and was cured by systemic antibiotic treatment with local disinfection. In this series, no patient had major complications such as soft tissue failure, structural failure, deep infection, permanent neurological deficit, or required amputation. Although three patients (33.3%) had a complication, none were major.

## Discussion

Limb salvage surgery has become the preferred practice versus amputation for osteosarcoma of the distal end of the femur since 1986^17^. However, unacceptable walking gait and poor function due to the LLD occur frequently in clinical practice. The consensus on the surgical indications of LLD is lacking, but it is generally believed that < 2 cm does not affect the patient's function, 2–4 cm can be corrected by shoe lift, and > 4 cm is suggested to be solved by different limb lengthening methods^18–20^. LLD has been one of the most challenging issues for limb salvage surgery with more patients with osteosarcoma achieving long-term survival^21–24^. The reasons for LLD can be biological reconstruction failure (bone absorption), prosthesis subsidence due to aseptic loosening, regular prosthesis replacement for immature patients, and so forth. We developed a novel staged lengthening strategy for LLD. While implementing this strategy, only an external fixator and standard static mega prosthesis (two-time honored procedures) were needed. Both hardware were easy to access, and the needed surgical techniques were quite mature. Our early results showed satisfactory LLD correction, favorable functional outcomes, and fewer complications.

### Limitations

This study had several limitations. First, our series of nine patients was small. More patients are needed to verify the functional outcomes, LLD correction, and complications. Second, this was a retrospective analysis of patients who received this lengthening strategy. A prospective study with a comparison cohort such as expandable prosthesis replacement would be preferred to compare this technique with other lengthening strategies. However, the unavailability of extendable prostheses in our country currently makes it impossible to perform such clinical studies currently. Nevertheless, we believe that our findings may provide initial evidence that this lengthening strategy has some merit and requires further exploration, even for patients with expandable prostheses available. We think that the preliminary presentation of this strategy may encourage other surgeons to attempt it in clinical practice. Finally, the mean follow-up of 20 months (3–70) (from the third surgery in stage 3) was insufficient to address the long-term complications and functional outcomes associated with this strategy. Nevertheless, we can assume that the long-term outcome should be as in reports using mega prosthesis since they are basically the same after LLD correction.

### Functional outcomes

The mean MSTS score of this series at the final follow-up was 96.3% (86.7%–100%), significantly superior to that before the lengthening. The MSTS function was comparable to that in other studies of regular mega-prosthesis reconstruction^25,26^, and even better than that in some previous reports^27–29^. While seeking the reasons for the functional score improvements, it was found that patients suffered greatly from LLD. Leg lengthening improved five of the subsets of MSTS score, including function, emotional acceptance, supports, walking, and gait. Although two patients with total femoral prosthesis replacement showed gluteal gait and limited walking ability, both were quite satisfied with the outcome of the lengthening procedure. Except for the patient with pathologic fractures, most of our patients wore shoe lifts before lengthening. However, 37.5% (3/8) of the patients developed prosthesis breakage, which made it impossible to continue wearing shoe lifts and hence surgery had to be performed. Besides, prosthesis breakage itself was believed to be associated with LLD. The ROM of the current series was lower than that of those who underwent regular mega-prosthesis replacement^30^. The long-term straight leg may contribute to ROM limitations. Although the designed mean duration from the first surgery to the third surgery (straight leg) was 6 months, it took as much as 12 months eventually due to COVID-19.

### LLD correction

The treatment of LLD in adult patients with sarcoma was rarely reported in the literature. Expandable prosthesis, which was designed for skeletally immature patients, was a method of compensating for the overgrowth of the contralateral limb. Only few cases were reported to use expandable prostheses in the adult population^12,13,20^. Sewell reported nine adults who received noninvasive expandable prostheses. The mean length gained was 56 mm (19–107), requiring a mean of nine lengthening episodes^12^. In a systematic review of expandable prostheses in skeletally immature patients^31^, the mean leg lengthening was 4.3 cm, with a mean of 4.4 lengthening procedures. However, the rate of LLD > 2 cm was 31% for patients over 16 years in age at the final follow-up. In our series, the leg length gain ranged from 3.6 cm to 15.6 cm with three surgeries. The mean LLD at the last follow-up was 0.3 cm, ranging from − 0.3 to 2.1 cm. The current lengthening strategy showed obvious advantages regarding the final LLD correction. Distraction osteogenesis using the external fixator has been reported to correct LLD^15,32^. However, the distraction was commonly performed on the tibia side, resulting in an elevated knee center compared with the contralateral limb. The impact of the knee center change on gait and function was unknown.

### Complications

The complication rate of expandable prostheses remains high. According to a recent systematic review of 292 patients^31^, the overall complication and revision rate was 43%, increasing to 59% and 89% in patients with more than 5 and 10 years of follow-up, respectively. In the current lengthening process, after the removal of the fixator, the final prosthesis was not introduced simultaneously to prevent the possible spread of infection from the pin sites to the prosthesis. Besides, about 2 cm overcorrection in stage 1 was recommended because acute shortening might be needed during the third surgery to release excessive soft tissue tension and cover the components. We believe that the aforementioned measures may contribute to the early favorable low complication rate. Long-term complications may be anticipated comparable with those of other static mega-prostheses.

In addition, the duration of the entire treatment phase was extended, with the period from Stage 2 to Stage 3 being particularly affected. This extension was primarily driven by the COVID-19 pandemic, which caused widespread disruptions and delays due to health and safety restrictions. A secondary factor contributing to the extended timeline, especially evident from Stage 2 to Stage 3, was the need for a cautious approach in managing patients with suboptimal soft tissue conditions after achieving the desired limb length. These combined circumstances, predominantly influenced by the pandemic, underscored our commitment to patient safety and treatment efficacy in a challenging environment.

## Conclusions

Managing LLD in patients with musculoskeletal tumors is still extremely challenging. Few effective and safe options are available. Surgical treatment mainly needs to restore the equal length of both limbs, including bone and soft tissue. This study proposed a method to restore the soft tissue length in the affected leg with an external fixator, while the bony length was restored with a standard static prosthesis. Both external fixator fixation and standard prosthesis plantation are mature techniques. The authors adopted staged surgery instead of directly placing the prosthesis after removing the external fixator to lower the potential complications related to the external fixator. During the lengthening stage, the prosthesis must be overcorrected by 2 cm. Further optimizing the lengthening procedure, shortening the total lengthening time, and strengthening the postoperative functional rehabilitation in the future are necessary.

In summary, we believe that staged lengthening and reconstruction with standard static prosthesis is a reasonable option for skeletally mature patients with unacceptable LLD.

## Data Availability

The datasets used and/or analysed during the current study available from the corresponding author on reasonable request.
